# Herbicide glyphosate efficiently inhibits growth of pathogenic *Prototheca* algae species, suggesting the presence of novel pathways for the development of anti-algal drugs

**DOI:** 10.1128/spectrum.02343-24

**Published:** 2025-01-27

**Authors:** Olga Makarova, Diana Steinke, Uwe Roesler

**Affiliations:** 1 Institute for Animal Hygiene and Environmental Health, Freie Universität Berlin 9166 , Berlin, Germany; 2 Unit of Veterinary Public Health and Epidemiology, University of Veterinary Medicine Vienna 27260 , Vienna, Austria; University of Saskatchewan, Saskatoon, Saskatchewan, Canada

**Keywords:** *Prototheca *species, protothecosis, fungal-like pathogens, anti-algal, glyphosate, shikimate pathway

## Abstract

*Prototheca* are ubiquitous algae and occasional pathogens of humans and animals. While rare, the infection is often fatal and treatment options are limited to antifungals with low efficiency. Here, using growth curve assays, we demonstrate that five pathogenic species of *Prototheca* (*P. blaschkeae, P. wickerhamii, P. cutis, P. ciferrii, P. bovis*) were fully inhibited by 50–100 μg/mL of herbicide glyphosate, suggesting novel pathways that can be considered for anti-algal drug development.

**IMPORTANCE:**

*Prototheca* are algae frequently found in the environment that occasionally cause infections in humans and animals. Although these infections are rare, they are often deadly for immunocompromised patients. Considering the rising ambient temperatures that promote algal bloom and a growing number of immunocompromised patients globally, such cases are likely to increase and will require efficient medications. Currently, the treatment is limited to antifungals that affect algal and animal membranes alike at concentrations close to toxic. Here, we hypothesized that targeting a pathway that is present in plants but not animals may be a new approach to the development of novel anti-algal compounds with high efficiency and lower toxicity. In this proof-of-principle study, we found that herbicide glyphosate, which targets the shikimate pathway found in plants but not in animals, efficiently inhibits all five tested pathogenic *Prototheca*, suggesting that the shikimate pathway may be a promising target for anti-algal drug development.

## OBSERVATION

**Fig 1:**
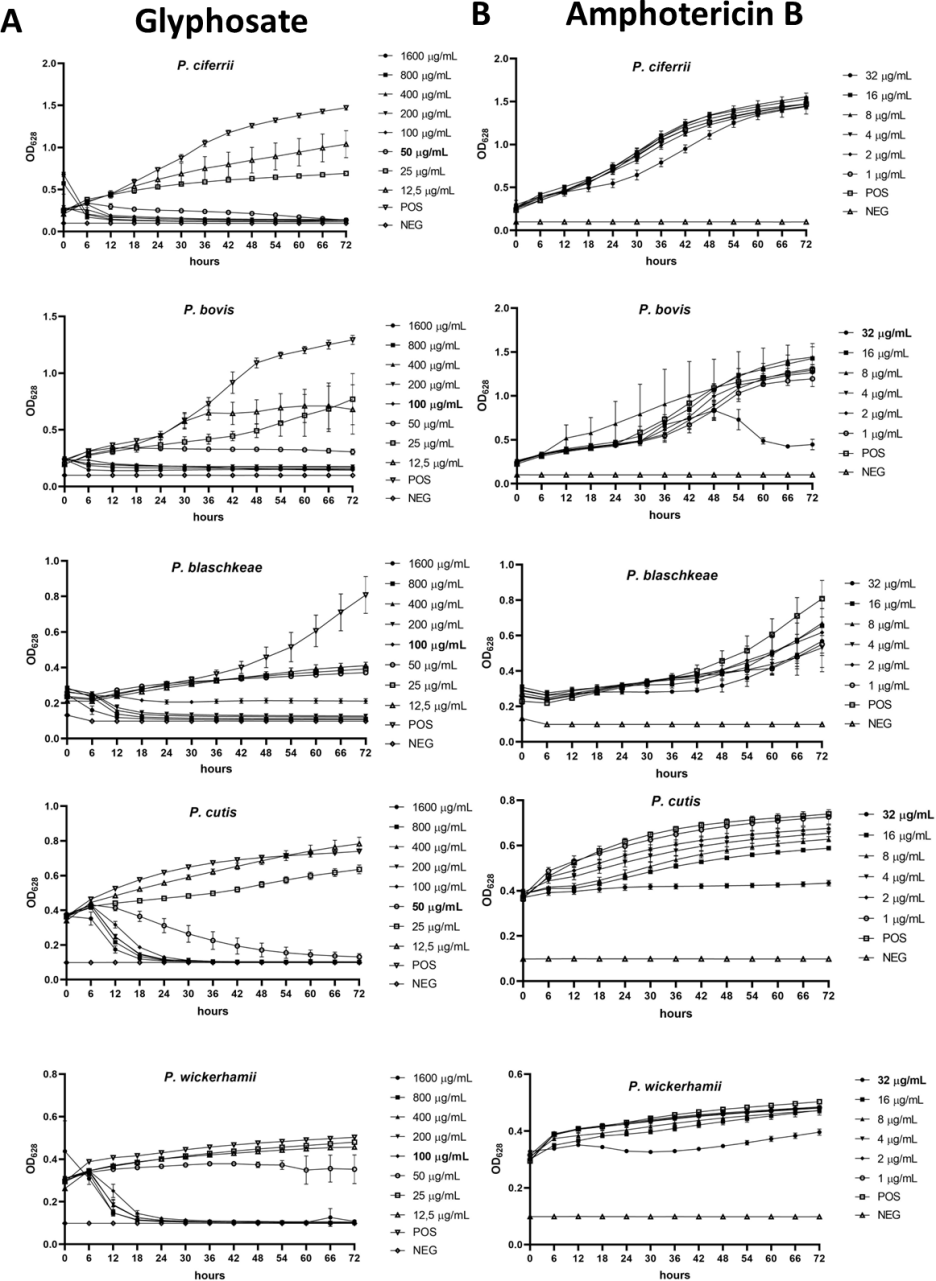
Growth curves of the five *Prototheca* species tested against glyphosate (**A**) and amphotericin B (**B**). POS, positive control (0 µg/mL glyphosate); NEG, negative control; OD_628_, optical density at 628 nm. Bars are ±SD of the mean. Inhibitory concentrations (determined as the lowest concentration at which OD_628_ values at the final 72 h time point were equal to or below those at time 0) are in bold.

**Fig 2:**
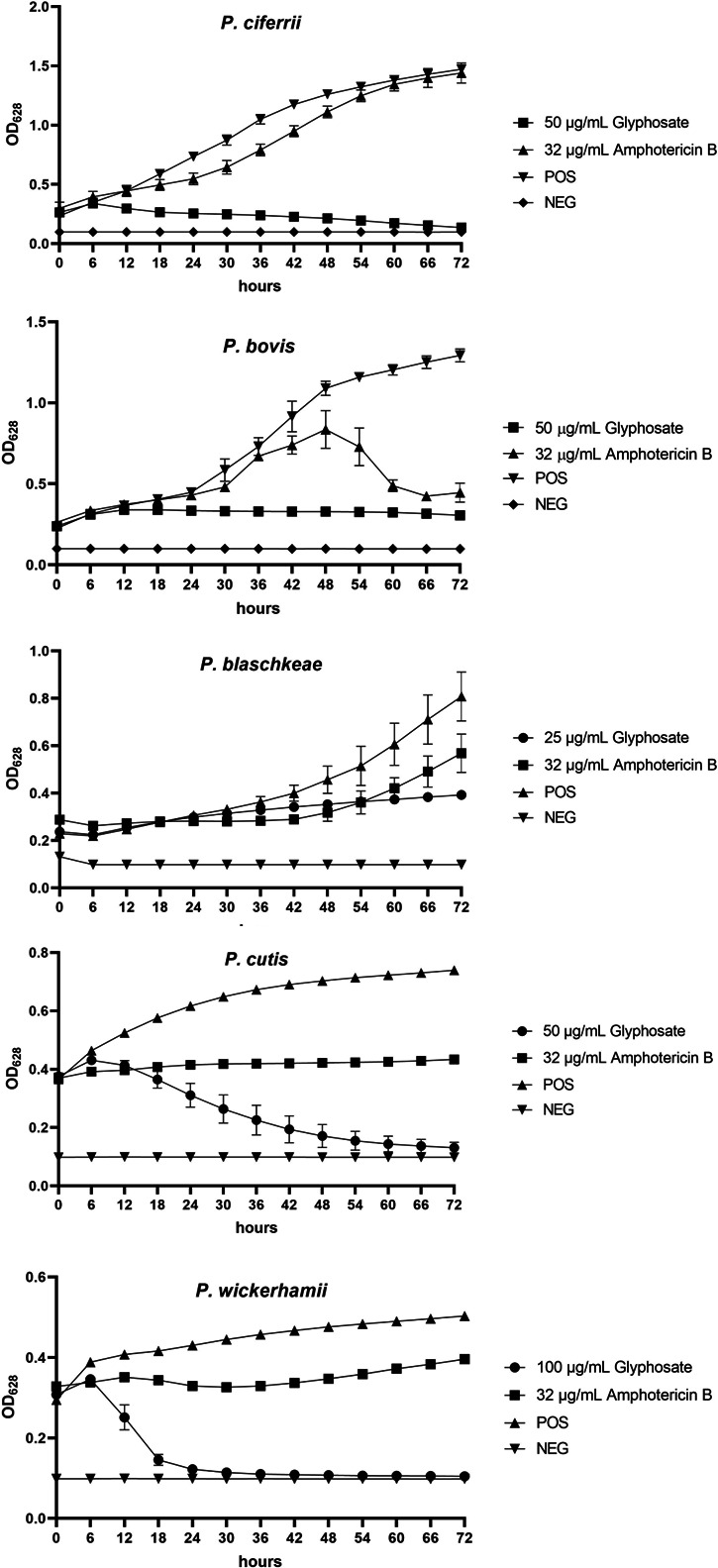
Growth curves of the five *Prototheca* species in the presence of glyphosate at inhibitory concentrations and amphotericin B at inhibitory concentrations (*P. cutis, P. wickerhamii,* and *P. bovis*) or the highest tested concentrations (*P. blaschkeae, P. ciferrii*). POS, positive control (0 µg/mL glyphosate); NEG, negative control; OD_628_, optical density at 628 nm. Bars are ±SD of the mean. Inhibitory concentrations were determined as the lowest concentration at which OD_628_ values at the final 72 h time point were equal to or below those at time 0.

*Prototheca* species are achlorophyllic algae from the family *Chlorellaceae*, closely related to green algae of the *Chlorella* genus (1). They are frequent colonizers of soil and aqueous environments, as well as animal intestines (2), and have been isolated from farms, pasture soils, and human sewage (1 and references therein). Several species have been described as opportunistic pathogens of humans and animals: *P. wickerhamii, P. cutis*, *P. bovis* (formerly *P. zopfii* genotype 2), *P. ciferrii* (formerly *P. zopfii* genotype 1), and *P. blaschkeae* (3–6). Although the infection in humans is considered to be rare and about the half of all cases have a cutaneous presentation, disseminated infection in immunocompromised patients is associated with a particularly poor outcome with over 50% death rate (7), and the cases have been increasing globally (8). Initially assumed to be yeasts, *Prototheca* infections are still treated with antifungals amphotericin B and azole drugs, which are often used at concentrations close to toxic (2). Therefore, more effective drugs are urgently needed. Indeed, several novel therapeutic options for protothecosis have been proposed, such as development of less toxic derivatives or formulations of amphotericin B (9, 10), re-purposing of existing antibiotics (11) and antifungals (12), nanoparticles (13), essential oils (14, 15), as well as a fungicide (16). Glyphosate is a popular herbicide that targets the shikimate pathway present in plants, unicellular parasites, fungi, and bacteria (17) and was also patented as an antimalarial compound (18). Notably, this biochemical pathway is absent in vertebrates, including humans, which results in low overall toxicity of glyphosate (19). Considering that protothecans are algae, we hypothesized that herbicide glyphosate may be efficient at supressing their growth. To test this, we performed growth curves of five *Prototheca* species in a range of glyphosate and amphotericin B concentrations. Briefly, strains were streaked from cryostocks on Sabouraud dextrose agar (SDA) (Oxoid, UK) and grown aerobically for 48–72 h. Throughout all experiments, *P. blaschkeae* P30*, P. wickerhamii* P4, and *P. cutis* DSM 22,084 P31 were incubated at 28°C, while *P. bovis* SAG 2021 P26 and *P. ciferrii* SAG2063 P23 were grown at 37°C. Individual single colonies were inoculated into 10 mL Sabouraud medium (Oxoid, UK) (three replicates per strain) and cultured for 48–72 h with shaking. When the cultures reached the optical density of 0.8 at 628 nm (OD_628_), they were diluted twofold with fresh Sabouraud medium, and 100 µL was used to inoculate 96-well polystyrene F-bottom plates (Sarstedt GmbH, Germany) containing 100 µL of the drug to achieve the final inoculum concentration of approximately 1–5 × 10^5^ CFU/mL. Additionally, cultures were serially diluted (10^1^–10^4^) and plated (100 µL) on SDA, incubated for 72 h and had their CFU/mL counted. Glyphosate, 40% aqueous solution (Sigma-Aldrich Chemie GmbH, Germany) was twofold diluted in Sabouraud medium in the range of 12.5–1,600 µg/mL. As glyphosate is known to acidify media at high concentrations, pH was controlled with pH indicator strips (Merck KGaA, Germany) and adjusted with 5 M NaOH to neutral, when necessary. Amphotericin B (E434-100 mg, Amresco Inc, USA) was diluted twofold in Sabouraud medium in the range of 1–32 µg/mL. Plates were incubated in a plate reader (Synergy HTX; BioTech Instruments, Germany), where the growth was followed at OD_628_ at 5 h intervals for 72 h. The OD_628_ values of six replicates for each time point were averaged and plotted using GraphPad Prism 8. GRcalculator (20) was used to calculate glyphosate’s half maximal inhibitory concentration (IC_50_), the concentration of drug when it produces its maximal effect (E_inf_) and area under the curve (AUC) values using the traditional sigmoid normal methodology. The growth curves revealed efficient inhibition of all tested *Prototheca* strains by glyphosate at the 50–100 µg/mL concentration range (determined as the lowest concentration at which OD_628_ values at the final 72 h time point was equal to or below those at time 0) (Fig. 1A; Table 1), which is consistent with the lethal concentrations of glyphosate (50–100 µg/mL) for several freshwater phytoplankton species, including *Chlorella* (21), and minimum inhibitory concentration (MIC) (97.5 µg/mL) for the unicellular green chlorophyte *Chlamydomonas reinhardtii* (22), and considerably lower than MIC for Enterobacteriaceae (10–80 mg/mL) (23, 24). The inhibitory concentrations of glyphosate were in the similar range of those for the antifungal amphotericin B (32 µg/mL) (Fig. 1B; Table 1), which is used for the treatment of protothecosis but showed more consistent inhibition among the tested *Prototheca* species and a lower tendency for regrowth than amphotericin B (Fig. 1 and 2). Drug efficacy parameters at the 72 h time point also showed a potent inhibition by glyphosate, with *P. blaschkeae*, *P. bovis*, and *P. ciferrii* displaying particularly low IC_50_ values (18.9, 19.4, and 20.7 µg/mL, respectively), while *P. cutis* and *P. wickerhamii* were somewhat less sensitive (35.2 and 60.8 µg/mL, respectively) (Table 2). Nonetheless, all tested species had an order of magnitude lower IC_50_ than those of *Chlorella* spp. (25). The inhibitory effects of glyphosate on *Prototheca* spp. may be explained by the presence of the shikimate pathway, the target of glyphosate (26, 27). Low acute toxicity of glyphosate (no observed adverse effect level [NOAEL] in dogs is 53 mg/kg bw per day) (28) is credited to the absence of this metabolic pathway in animals. Conversely, the toxicity of amphotericin B, the front-line drug for treatment of human protothecosis, is attributed to its effects on mammalian membranes that contain sterols (the therapeutic target for amphotericin B), which limits the administration dose to 0.7–1 mg/kg/day (29). Our proof-of-principle study is the first to our knowledge to investigate the effects of a herbicide on pathogenic *Prototheca* spp. and suggests the presence of biochemical pathways that may be a promising target for the development of anti-algal drugs with low toxicity in animal cells. Future studies are needed to determine the exact mechanism of action to facilitate the design of target-specific molecules.

**TABLE 1:** Minimum inhibitory concentration for glyphosate and amphotericin B derived from the growth curves and determined as the lowest concentration at which optical density values at the 72 h time point were equal to or below those at time 0

Species	Glyphosate, µg/mL	Amphotericin B, µg/mL
*P. blaschkeae*	100	>32
*P. cutis*	50	32
*P. wickerhamii*	100	32
*P. ciferrii*	50	>32
*P. bovis*	100	32

**TABLE 2:** Drug efficacy parameters at the 72 h time point for glyphosate, expressed in µg/mL

Species	IC_50_	E_inf_	AUC	*P*-value	R^2^
*P. blaschkeae*	18.9	0.0412	0.593	0.0142	0.914
*P. cutis*	35.2	0.13	0.659	0.000401	0.992
*P. wickerhamii*	60.8	0.195	0.851	0.00121	0.987
*P. ciferrii*	20.7	0.0702	0.346	0.00755	0.965
*P. bovis*	19.4	0.0944	0.5	0.0355	0.842

## SUPPLEMENTAL MATERIAL

Supplemental figures spectrum.02343-24-s0001.pdf Individual figures that were used to prepare composite figures 1 and 2.

ASM does not own the copyrights to Supplemental Material that may be linked to, or accessed through, an article. The authors have granted ASM a non-exclusive, world-wide license to publish the Supplemental Material files. Please contact the corresponding author directly for reuse.
