# Supplementary material for: Herbicide glyphosate efficiently inhibits growth of pathogenic Prototheca algae species, suggesting the presence of novel pathways for the development of anti-algal drugs
Source: Microbiol Spectr. 2025 Jan 27;13(3):e02343-24. doi: 10.1128/spectrum.02343-24 (PMC11878087; doi:10.1128/spectrum.02343-24)
Supplement: Supplemental figures — Individual figures that were used to prepare composite figures 1 and 2. [file spectrum.02343-24-s0001.pdf]

Supplementary material
for the manuscript

"Herbicide glyphosate efficiently inhibits growth of pathogenic Prototheca algae species, suggesting the presence of novel pathways for the development of anti-algal drugs"
by

Olga Makarova[a,b], Diana Steinke[a], Uwe Roesler[a]

[a]Institute for Animal Hygiene and Environmental Health, Freie Universität Berlin, Berlin, Germany
[b]Unit of Veterinary Public Health and Epidemiology, University of Veterinary Medicine Vienna, Vienna, Austria

Figure Legends:
Growth curves of the five *Prototheca* species tested against glyphosate and amphotericin B. POS, positive control (0 µg/mL glyphosate); NEG, negative control; OD_{628}, optical density at 628 nm. Bars are ±SD of the mean. Inhibitory concentrations (determined as the lowest concentration at which OD_{628} values at the final 72 hours time point were equal to or below those at time 0) are in bold.

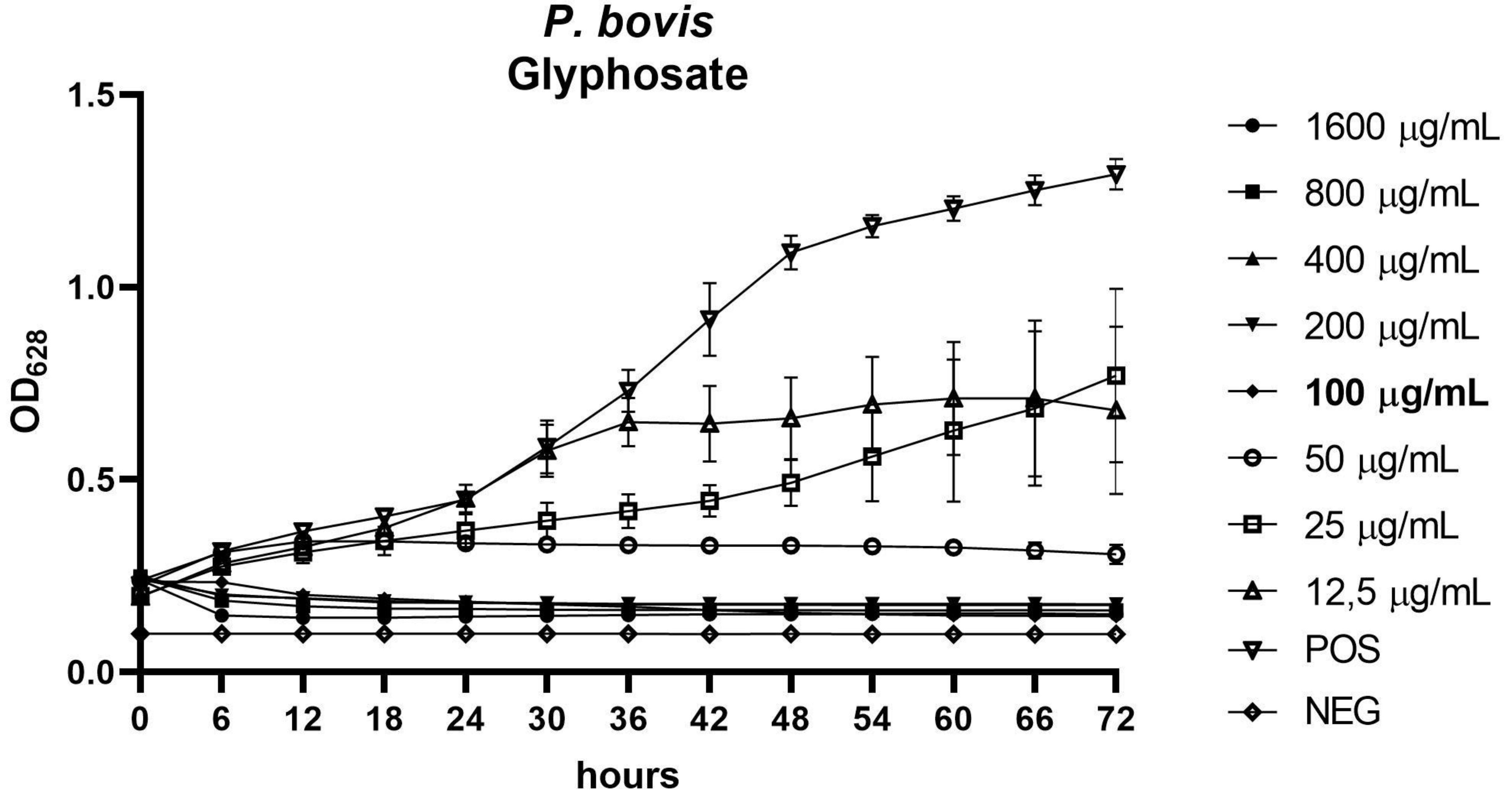

P. bovis
Glyphosate
1.5
1.0
0.5
0.0
OD628
0
6
12
18
24
30
36
42
48
54
60
66
72
hours
1600 µg/mL
800 µg/mL
400 µg/mL
200 µg/mL
100 µg/mL
50 µg/mL
25 µg/mL
12,5 µg/mL
POS
NEG

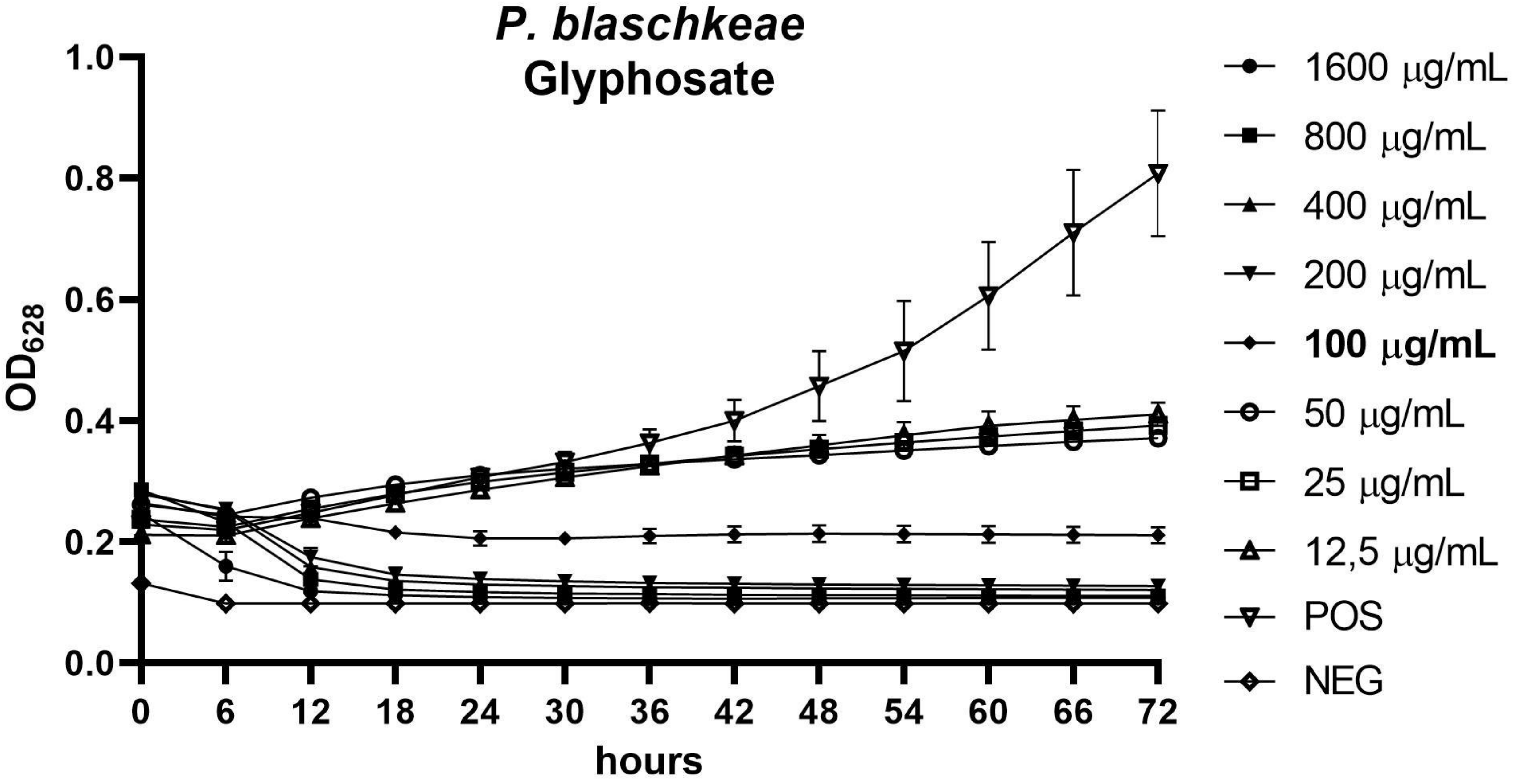
P. blaschkeae
Glyphosate
OD628
hours
0.0
0.2
0.4
0.6
0.8
1.0
0
6
12
18
24
30
36
42
48
54
60
66
72
1600 µg/mL
800 µg/mL
400 µg/mL
200 µg/mL
100 µg/mL
50 µg/mL
25 µg/mL
12,5 µg/mL
POS
NEG

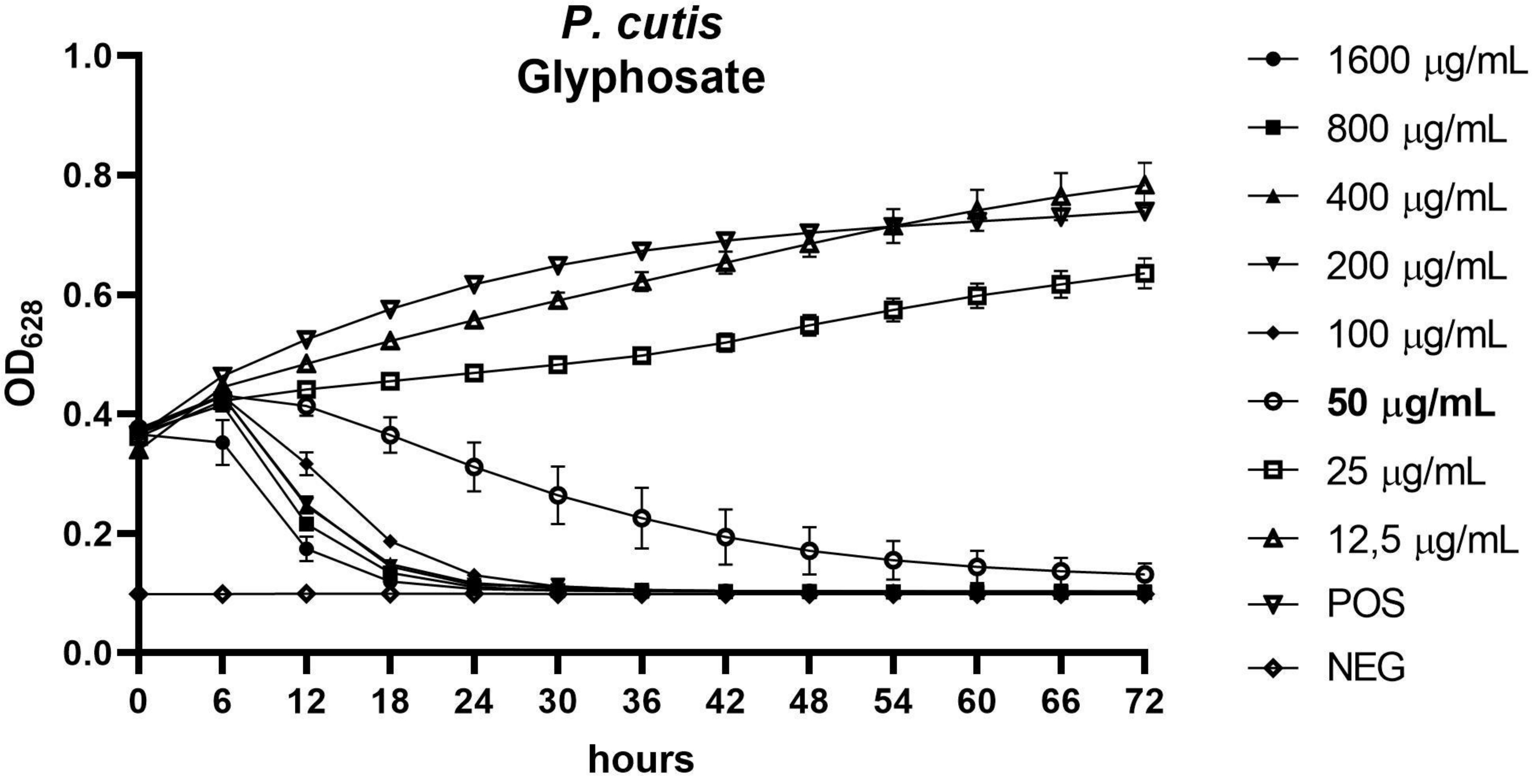
P. cutis
Glyphosate
OD628
1.0
0.8
0.6
0.4
0.2
0.0
0
6
12
18
24
30
36
42
48
54
60
66
72
hours
1600 μg/mL
800 μg/mL
400 μg/mL
200 μg/mL
100 μg/mL
50 μg/mL
25 μg/mL
12,5 μg/mL
POS
NEG

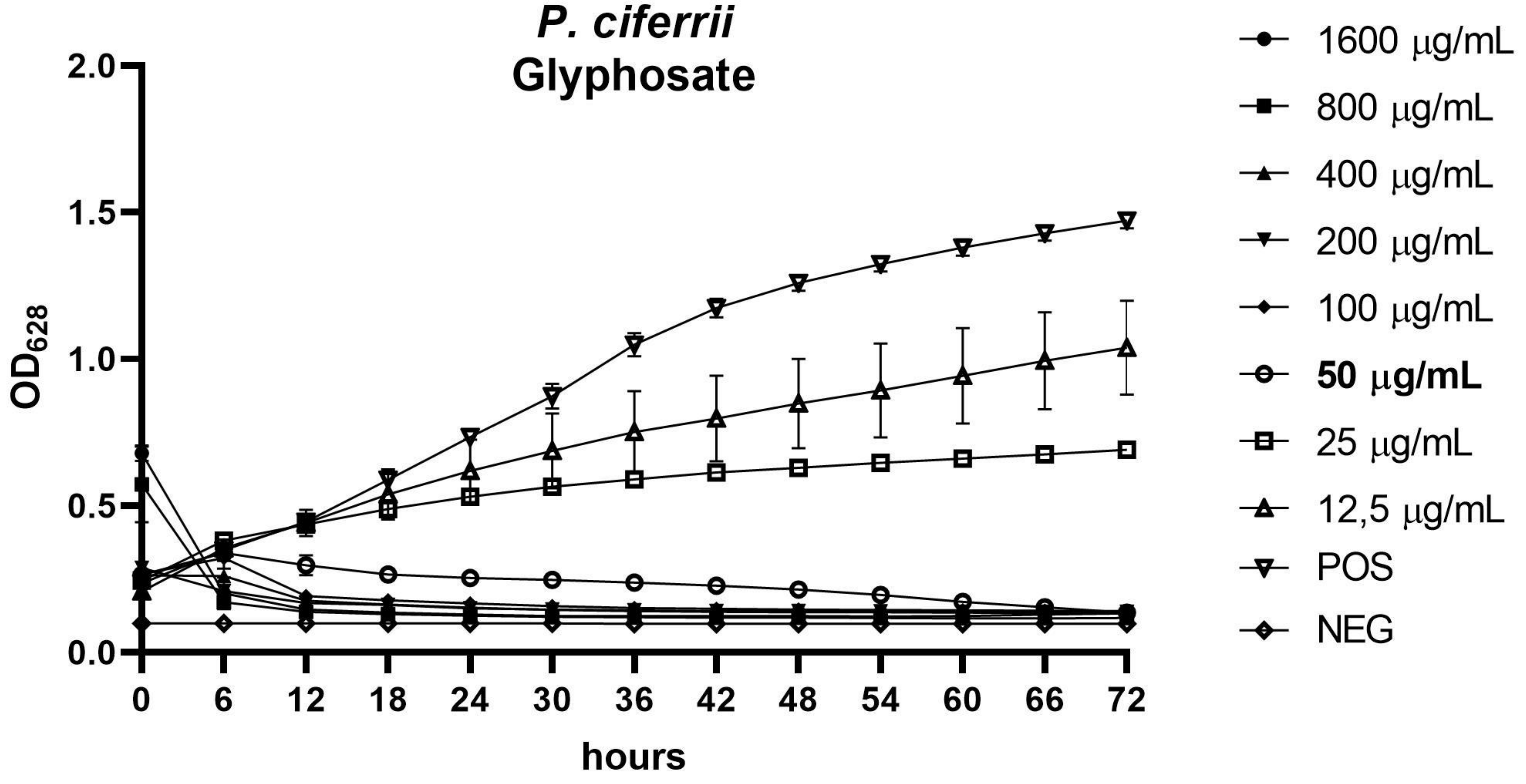

P. ciferrii
Glyphosate
OD628
2.0
1.5
1.0
0.5
0.0
0
6
12
18
24
30
36
42
48
54
60
66
72
hours
1600 µg/mL
800 µg/mL
400 µg/mL
200 µg/mL
100 µg/mL
50 µg/mL
25 µg/mL
12,5 µg/mL
POS
NEG

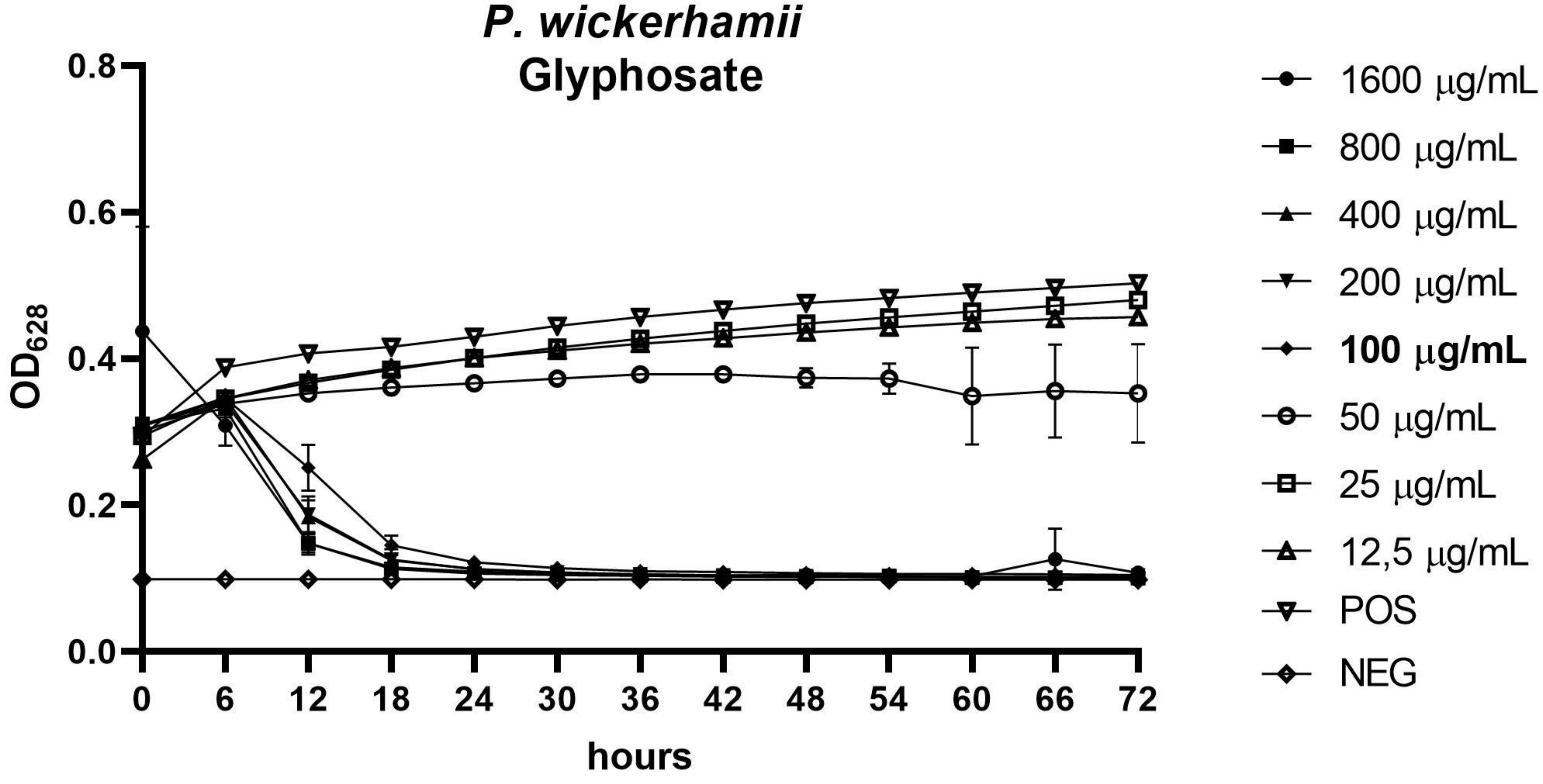

P. wickerhamii
Glyphosate
0.8
0.6
0.4
0.2
0.0
OD628
0
6
12
18
24
30
36
42
48
54
60
66
72
hours
1600 μg/mL
800 μg/mL
400 μg/mL
200 μg/mL
100 μg/mL
50 μg/mL
25 μg/mL
12,5 μg/mL
POS
NEG

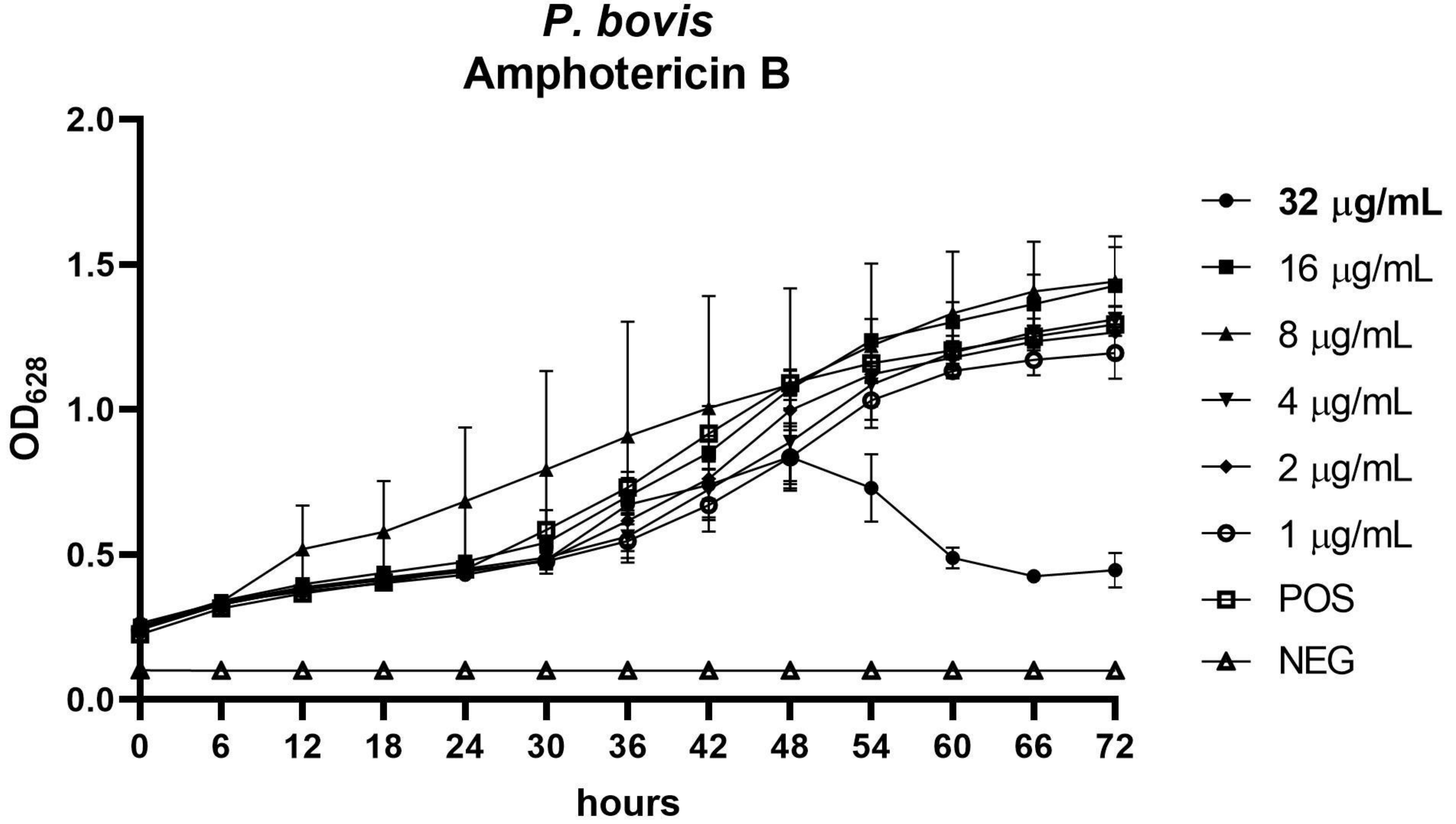

P. bovis
Amphotericin B
2.0
1.5
1.0
0.5
0.0
OD628
0
6
12
18
24
30
36
42
48
54
60
66
72
hours
32 μg/mL
16 μg/mL
8 μg/mL
4 μg/mL
2 μg/mL
1 μg/mL
POS
NEG

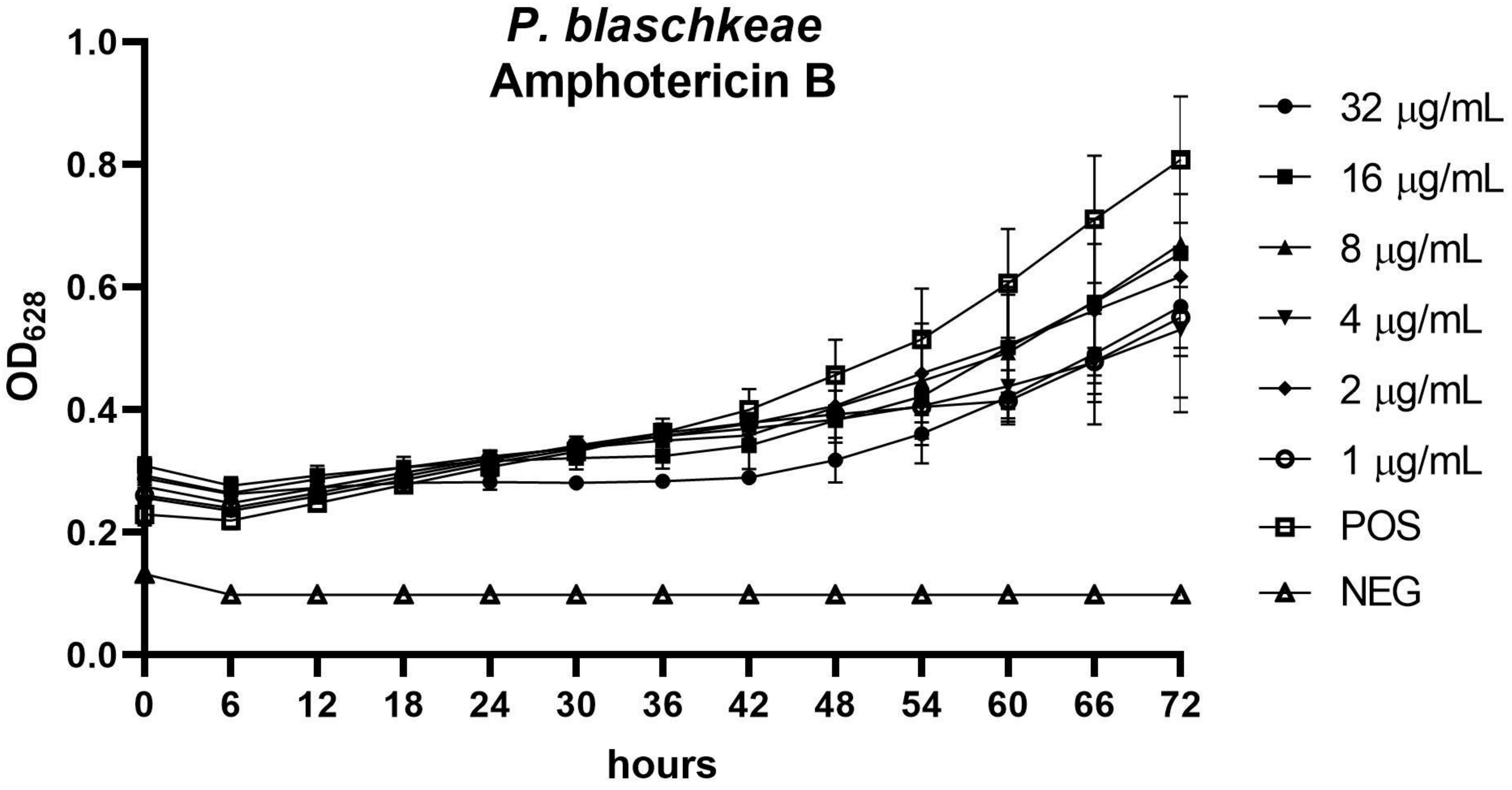

P. blaschkeae
Amphotericin B
1.0
0.8
0.6
0.4
0.2
0.0
OD628
0
6
12
18
24
30
36
42
48
54
60
66
72
hours
32 μg/mL
16 μg/mL
8 μg/mL
4 μg/mL
2 μg/mL
1 μg/mL
POS
NEG

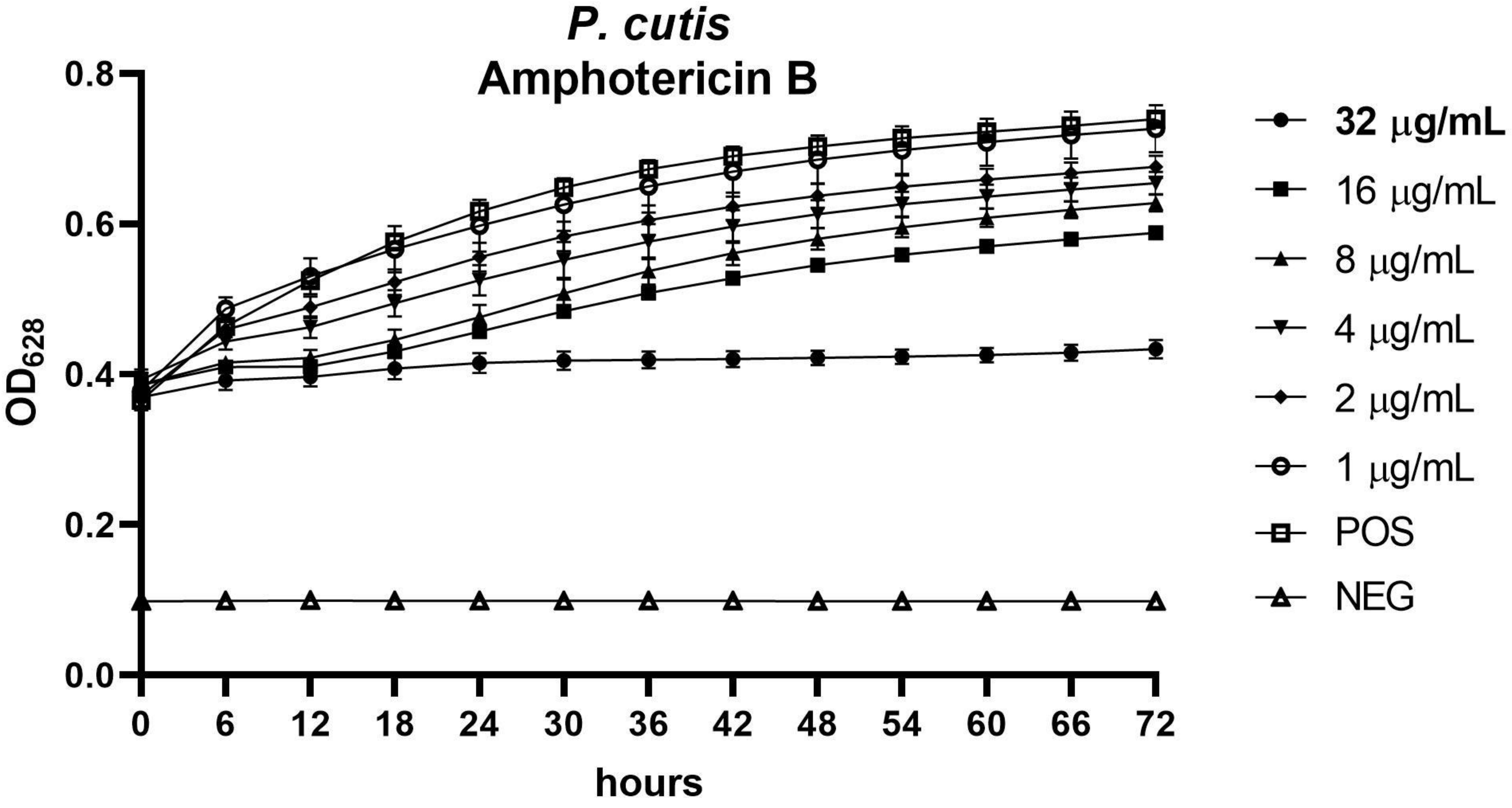

P. cutis
Amphotericin B
0.8
0.6
0.4
0.2
0.0
OD_{628}
0
6
12
18
24
30
36
42
48
54
60
66
72
hours
32 μg/mL
16 μg/mL
8 μg/mL
4 μg/mL
2 μg/mL
1 μg/mL
POS
NEG

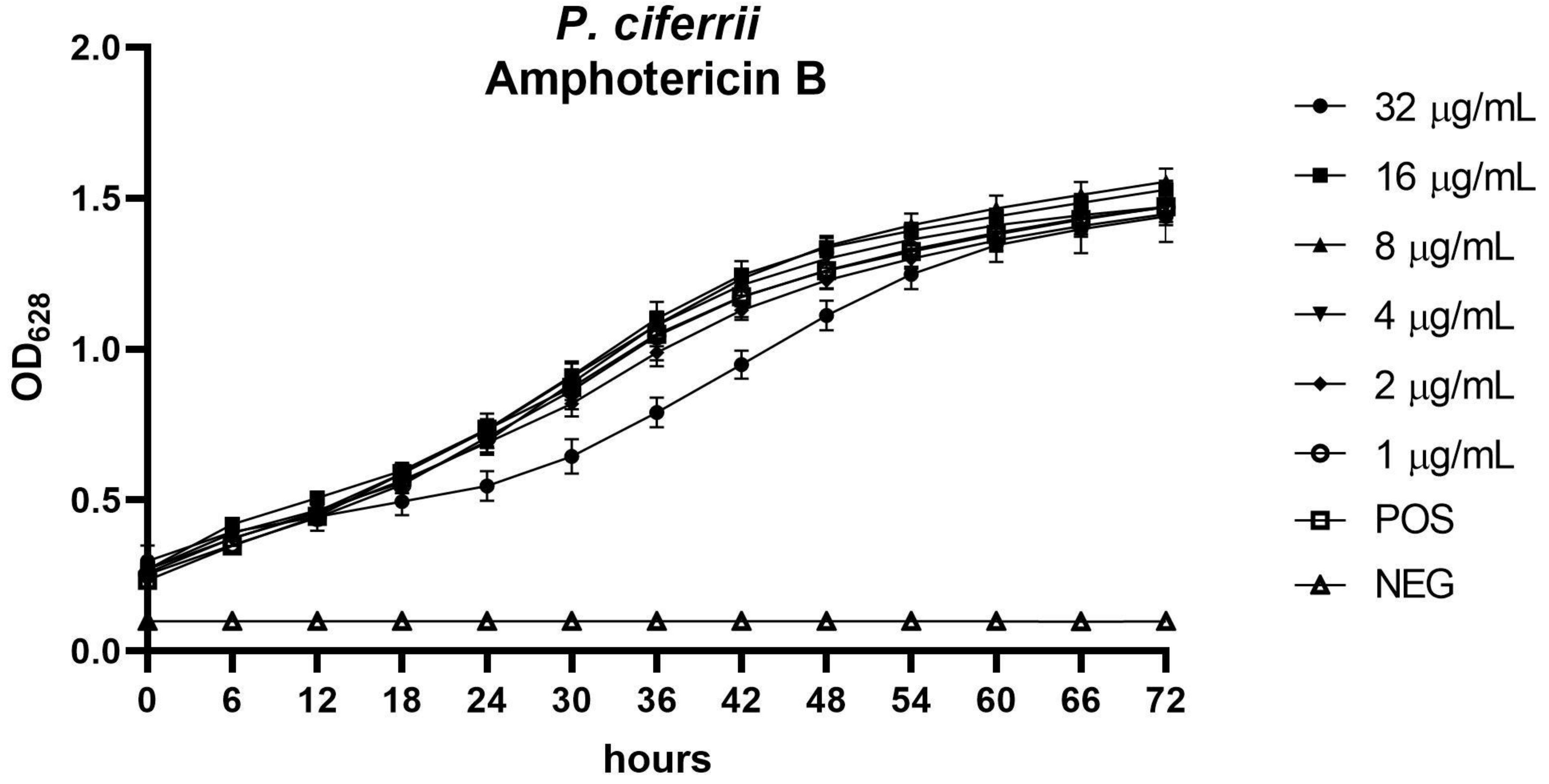

P. ciferrii
Amphotericin B
OD628
hours
2.0
1.5
1.0
0.5
0.0
0
6
12
18
24
30
36
42
48
54
60
66
72
32 μg/mL
16 μg/mL
8 μg/mL
4 μg/mL
2 μg/mL
1 μg/mL
POS
NEG

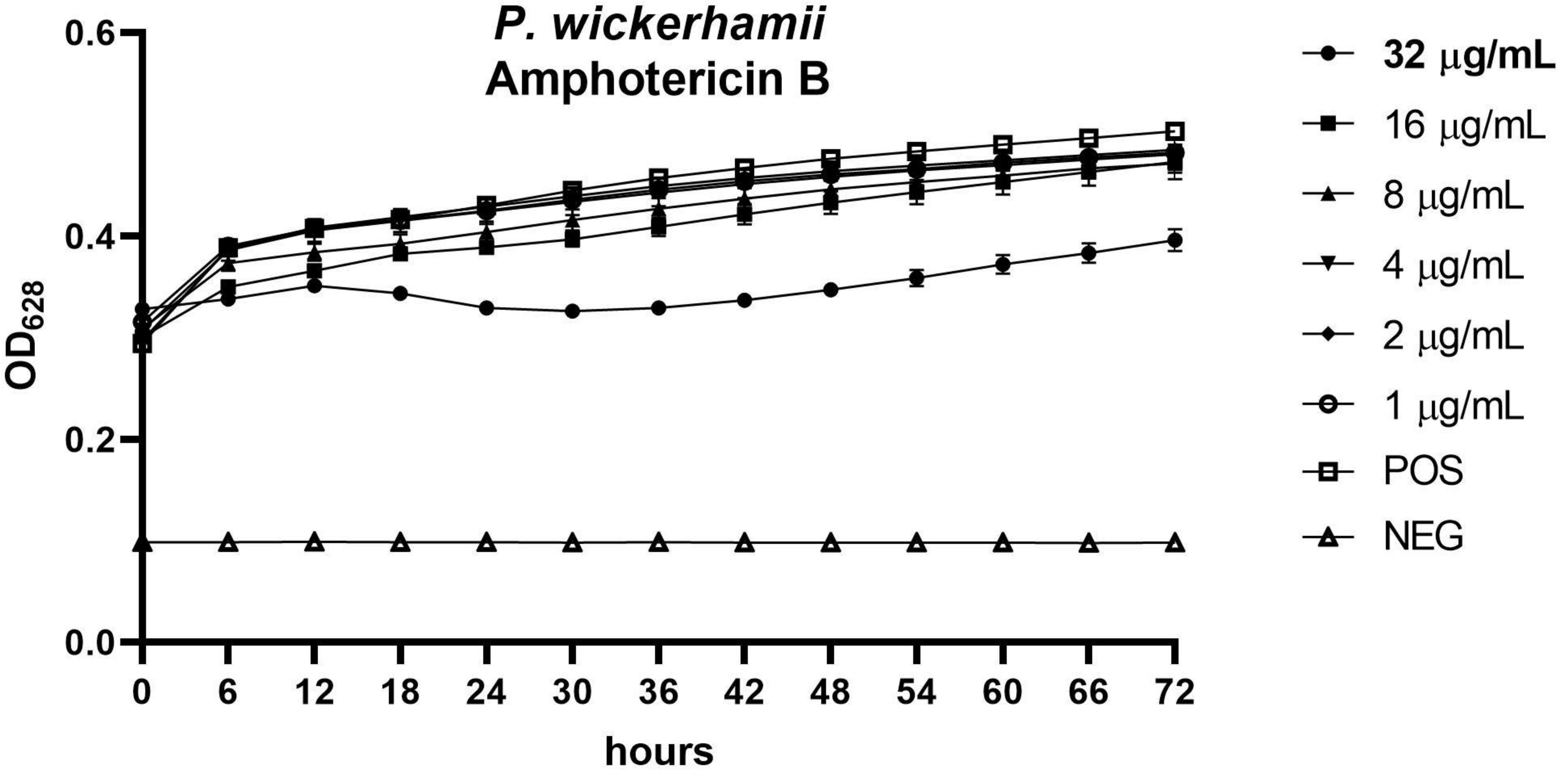

P. wickerhamii
Amphotericin B
0.6
0.4
0.2
0.0
OD628
0
6
12
18
24
30
36
42
48
54
60
66
72
hours
32 μg/mL
16 μg/mL
8 μg/mL
4 μg/mL
2 μg/mL
1 μg/mL
POS
NEG

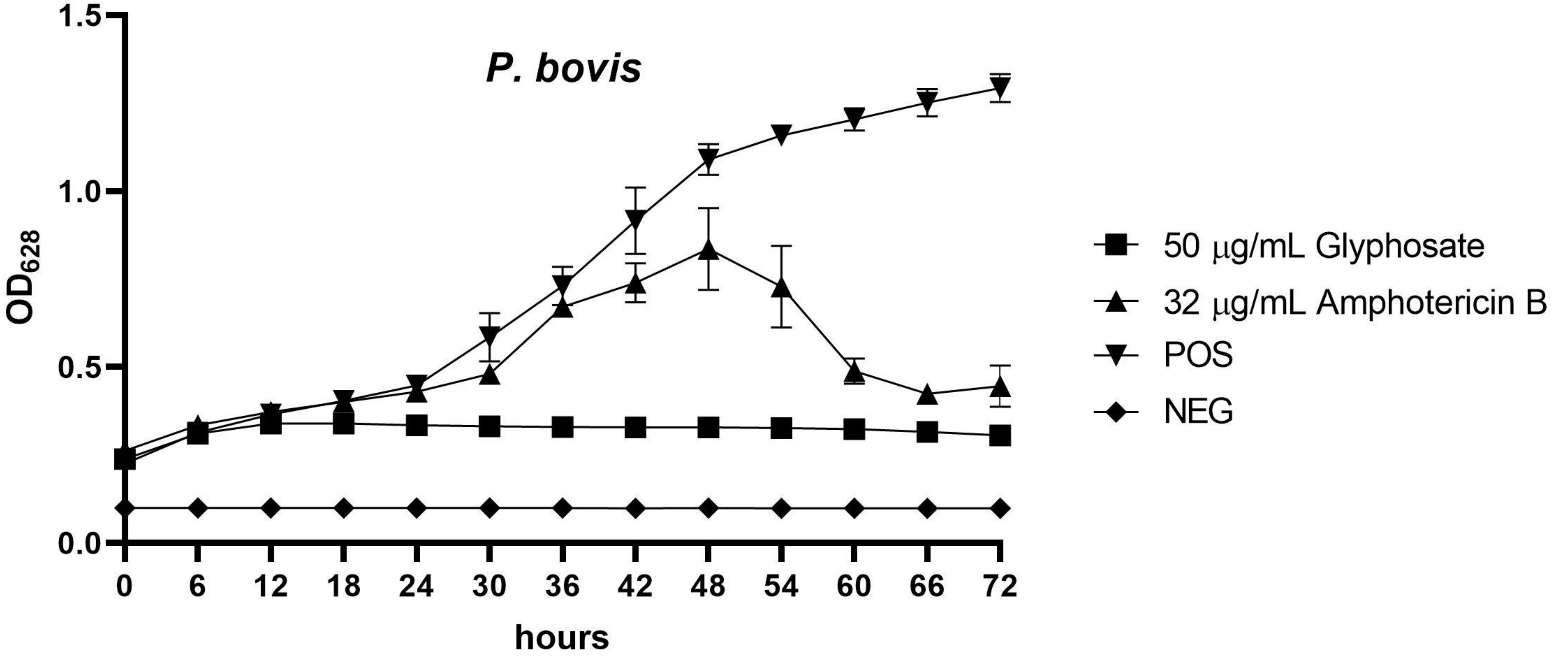
P. bovis
1.5
1.0
0.5
0.0
OD628
0
6
12
18
24
30
36
42
48
54
60
66
72
hours
50 μg/mL Glyphosate
32 μg/mL Amphotericin B
POS
NEG

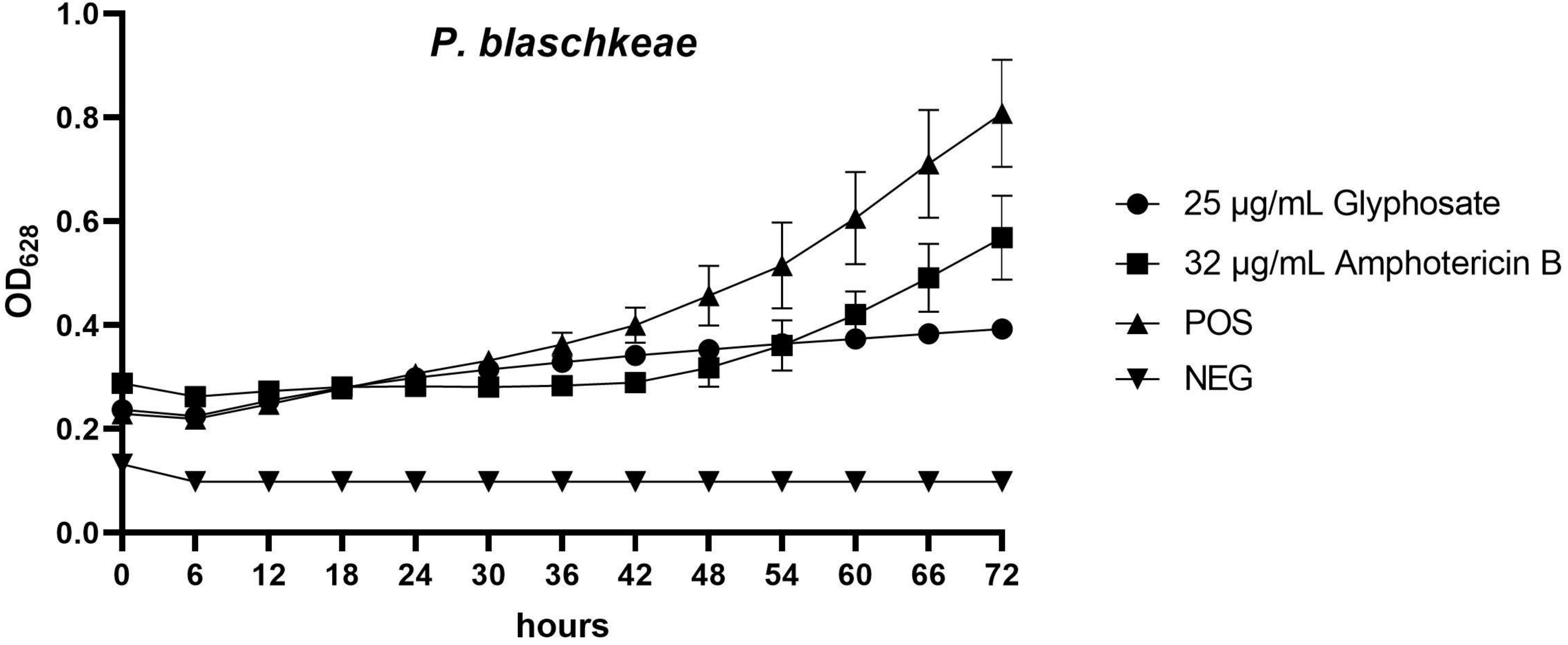

P. blaschkeae
OD628
hours
1.0
0.8
0.6
0.4
0.2
0.0
0
6
12
18
24
30
36
42
48
54
60
66
72
25 µg/mL Glyphosate
32 µg/mL Amphotericin B
POS
NEG

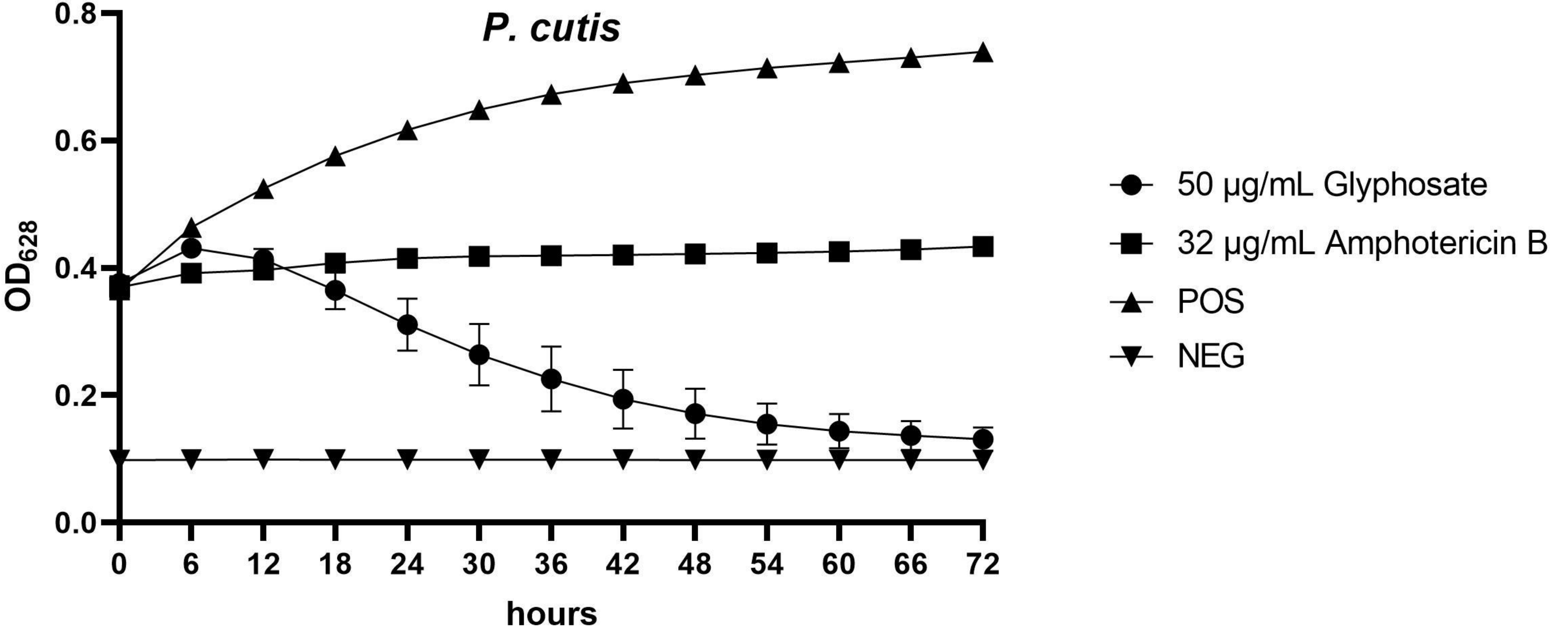
P. cutis
0.8
0.6
0.4
0.2
0.0
OD628
0
6
12
18
24
30
36
42
48
54
60
66
72
hours
50 µg/mL Glyphosate
32 µg/mL Amphotericin B
POS
NEG

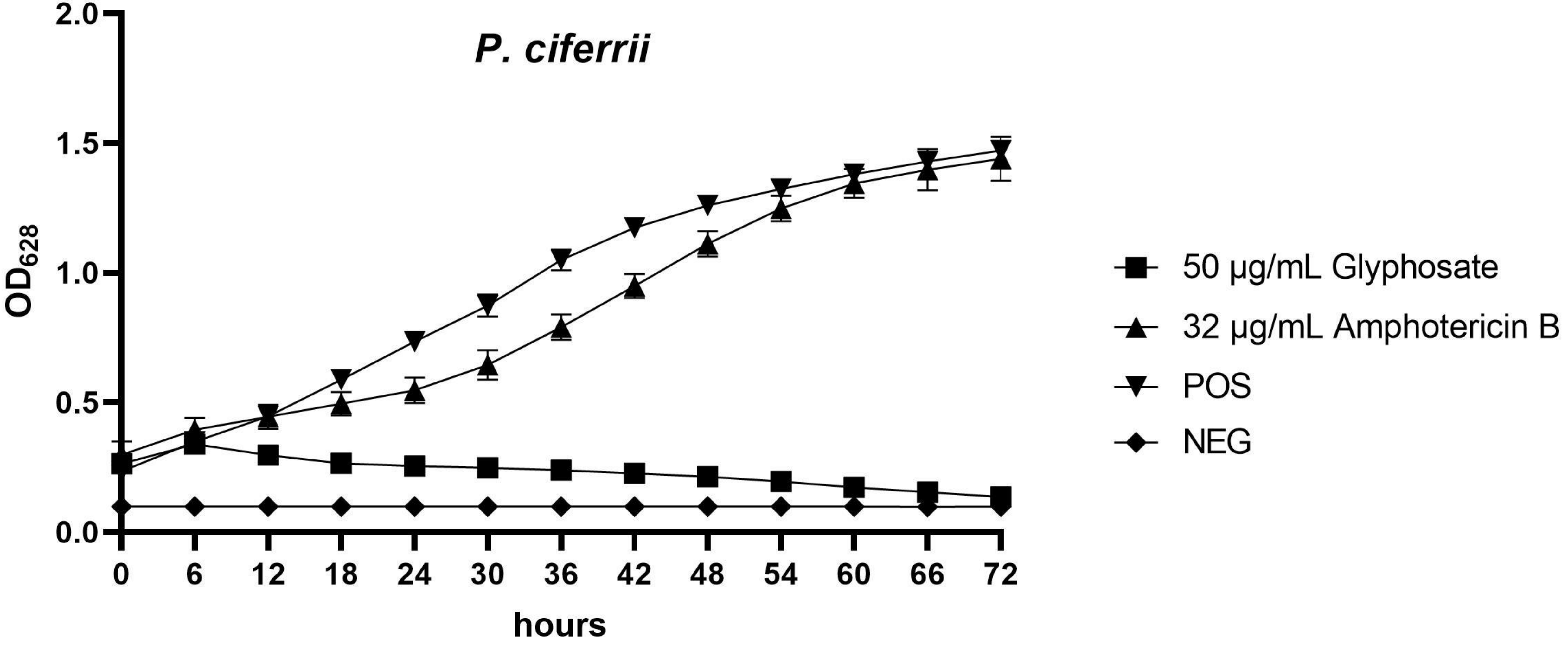
P. ciferrii
OD628
2.0
1.5
1.0
0.5
0.0
0
6
12
18
24
30
36
42
48
54
60
66
72
hours
50 µg/mL Glyphosate
32 µg/mL Amphotericin B
POS
NEG

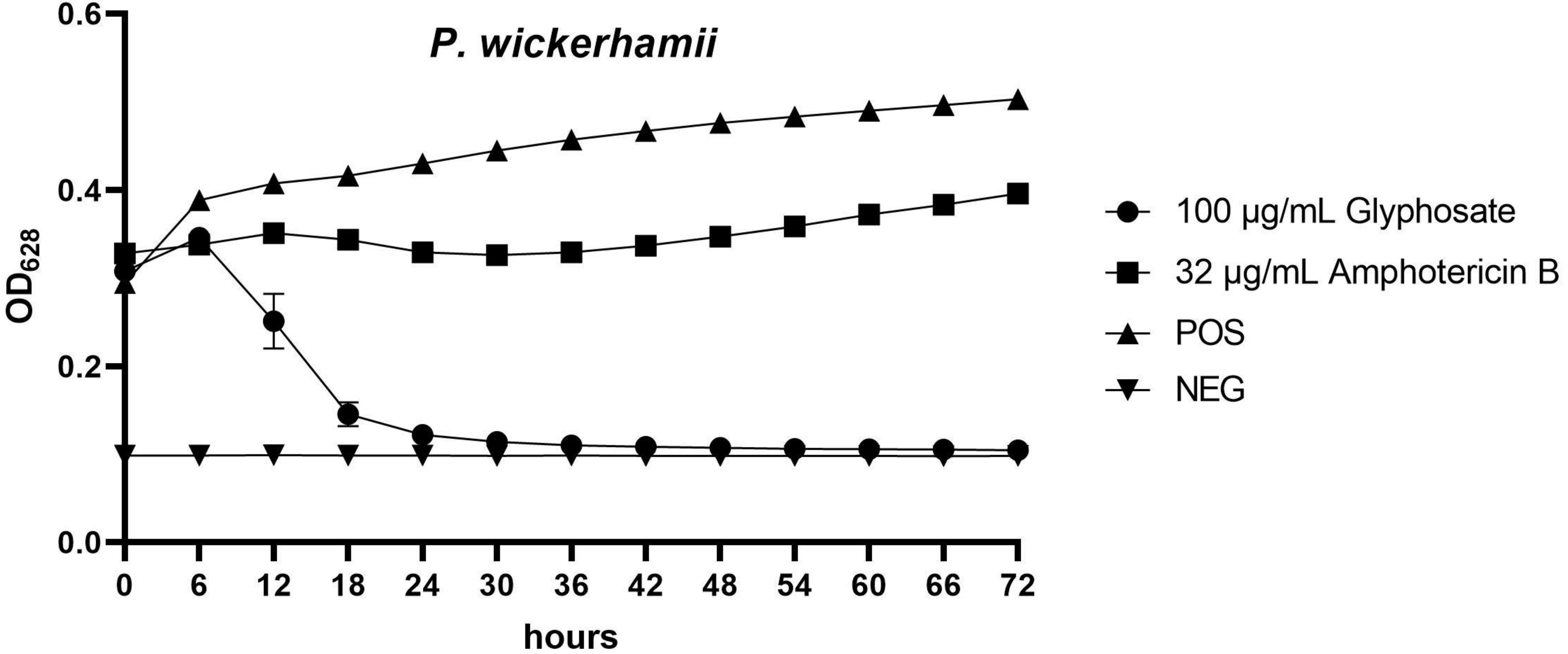

P. wickerhamii
0.6
0.4
0.2
0.0
OD628
0
6
12
18
24
30
36
42
48
54
60
66
72
hours
100 µg/mL Glyphosate
32 µg/mL Amphotericin B
POS
NEG
